# Lung Cancer with Brain Metastases Treated with Radiotherapy Followed by Bevacizumab Maintenance: A Case Report

**DOI:** 10.31729/jnma.7400

**Published:** 2022-06-30

**Authors:** Sajjad Ahmed Khan, Sakshyam Gautam, Sulav Sapkota

**Affiliations:** 1 Birat Medical College Teaching Hospital, Biratnagar, Morang, Nepal; 2 Department of Medical Oncology, Birat Medical College Teaching Hospital, Biratnagar, Morang, Nepal

**Keywords:** *bevacizumab*, *brain metastases*, *radiotherapy*

## Abstract

Brain metastases in a patient with non-small cell lung cancer carry a grave prognosis. Without effective interventions, the average survival rate is 6 months. Here, we present a case of a 59-year-old male with non-small cell lung cancer and multiple brain metastases treated with radiotherapy followed by bevacizumab maintenance with prolonged survival. There are limited studies establishing the efficiency and toxicity profile of anti-vascular endothelial growth factors for brain metastases. This reported case had a remarkable response with marked clinical and radiological improvement along with a tolerable toxicity profile. Showing the extent of effectiveness and efficacy of anti-vascular endothelial growth factor agents in the case of lung cancer with brain metastases is the main motto of our study.

## INTRODUCTION

Palliative care in a patient with brain metastases secondary to lung cancer is a topic of great concern in medical oncology.^1^ There is no definitive cure for the disease. The development of effective therapies against brain metastasis is currently hindered by limitations in our understanding of the molecular mechanisms driving it.^2^ Vascular Endothelial Growth Factor-A (VEGF-A) is a key regulator of tumour-induced angiogenesis and essential for tumour growth and distant tumour spread.^3^ Thus, VEGF can be a potential target to limit distant spread of the tumour. Here, we present a case of brain metastases secondary to lung cancer treated with an anti-VEGF agent.

## CASE REPORT

A 59-year-old gentleman came up with a chief complaint of haemoptysis on 30^th^ December, 2020. There was no history of fever, shortness of breath, recent weight loss, or weakness. Computed Tomography (CT) chest was done and findings were suggestive of malignancy involving the inferior lobe of the left lung, which was further confirmed on biopsy. Investigation for Epidermal Growth Factor Receptor (EGFR) status was done which turned out to be negative. There was no history of any chronic illness in the past but family history is significant for lung cancer in his uncle, grandfather who died of squamous cell carcinoma of the skin and his brother who died of colon carcinoma.

**Figure 1:**
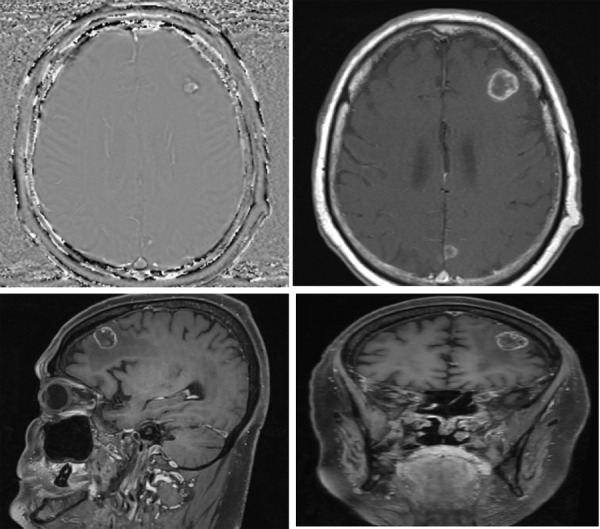
CT scan of the head shows brain metastases anteriorly in the left frontal lobe and in the central occipital lobe, prior to chemotherapy.

Following the diagnosis, surgical resection of the cancerous part was done. After surgery, the patient was given 6 cycles of taxane and platinum-based chemotherapy and he was doing apparently well for 4 months after surgery, when he started experiencing dizziness, syncopal attack, and tremors of the extremities, up to five episodes per day. The CT scan of the abdomen was done which revealed metastases to adrenal glands. CT head was done and metastatic foci in the anterior part of the left frontal lobe and occipital lobe were detected (Figure 1).

**Figure 2:**
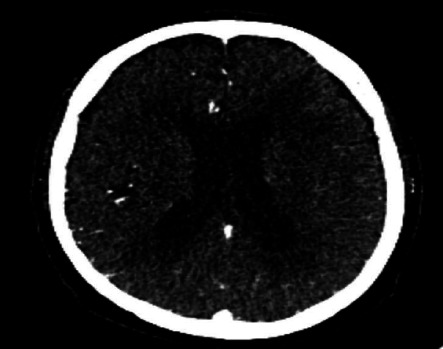
CT scan of the brain done after a year of treatment showing a marked decreased in vasogenic oedema and malignant changes.

Brain metastases were treated with radiotherapy followed by monthly bevacizumab maintenance-based chemotherapy. Marked improvement was noticed in the patient's clinical status, and features of brain metastases like dizziness and the syncopal attack had completely resolved. Further, a repeated CT scan of the head was done which showed a decrease in vasogenic oedema compared to the pre-interventional state (Figure 2).

## DISCUSSION

Even with emerging systemic therapies, having brain spreads with advanced lung cancer usually carries a poor prognosis and high morbidity. Treating a case of brain metastasis is a great challenge due to the lack of knowledge of molecular biology involved in it. Nevertheless, efforts are made to study the genetic alterations and environmental factors which predispose a tumour to undergo brain metastasis.

Hypoxia-inducible Factor-1 (HIF-1) is a master transcription factor that plays a central role in the hypoxic expression of various genes. Vascular endothelial growth factor, a known target gene of HIF-1a, has been shown to be induced by hypoxia through a HIF-1 a-independent pathway.^4^ Vascular Endothelial Growth Factor Receptor 2 (VEGFR2) is a 151-kDa member of the Receptor Tyrosine Kinase (RTK) family. This receptor is expressed on the surface of endothelial cells and controls angiogenesis, the formation of new blood vessels from existing vasculature, as well as vasculogenesis, the de novo formation of new blood vessels in tissues. Thus, VEGFR2 plays a critical role in human development and in cancer progression and is a valuable drug target. Indeed, therapies that inhibit VEGFR2 and angiogenesis would be applicable to many solid tumours, which need oxygen to grow.^5^

According to national comprehensive cancer network (NCCN) guidelines 2021, first-line treatment of driver mutation includes the use of EGFR inhibitor line erlotinib which may be combined with bevacizumab, a VEGF antagonist for a few years followed by a biopsy, if cancer continues to grow.^6^ However, in our case EGFR mutation was negative.

The symptoms of brain metastases are very nonspecific ranging from mild dizziness and tremors to syncopal attack. Unfortunately, till now, we do not have any definitive cure for the condition and palliative care to ease the life of the patient as well as prolong survivability is one of the evolving topics in medical oncology. A study was done in Italy among 39 patients who had brain metastases secondary to nonsmall cell lung cancer and were treated with injection pemetrexed, among which cerebral radiologic benefits were observed in 32 patients (82%) while seven of them showed progressive disease, with overall median survival rate 10 months.^7^

A prospective observational study done in a lung cancer treatment unit in an academic medical institute in Eastern Nepal concludes treatment of cancer in Nepal is limited by a lack of adequately trained surgeons, availability of diagnostic and treatment modalities and the study further emphasises the urgent need to train manpower and subsidise treatment of lung cancer in some form in our country.^8^ Even at the global level, studies regarding the treatment of lung cancer with brain metastases are very limited. Meta-analysis and systematic reviews did compare the efficacy and safety of different treatment modalities in these groups of patients but do not have very conclusive findings. So far now, there is no established way of dealing with the situation. In our reported case, both clinical and radiological improvements were seen in the patient on bevacizumab maintenance and his current health status seems to be better than before.
